# Development and Testing of an Intelligent Pain Management System (IPMS) on Mobile Phones Through a Randomized Trial Among Chinese Cancer Patients: A New Approach in Cancer Pain Management

**DOI:** 10.2196/mhealth.7178

**Published:** 2017-07-25

**Authors:** Yunheng Sun, Feng Jiang, Juan J Gu, Y Ken Wang, Hongwei Hua, Jing Li, Zhijun Cheng, Zhijun Liao, Qian Huang, Weiwei Hu, Gang Ding

**Affiliations:** ^1^ Xinhua Hospital Affiliated to Shanghai Jiao Tong University School of Medicine Shanghai China; ^2^ Xinhua Hospital Affiliated to Shanghai Jiao Tong University School of Medicine, Chongming Branch Shanghai China; ^3^ Lymphoma Translational Research Laboratory Department of Medicine Roswell Park Cancer Institute Buffalo, NY United States; ^4^ Division of Management and Education University of Pittsburgh at Bradford Bradford, PA United States

**Keywords:** cancer pain, intelligent pain management system, smart phone, intervention

## Abstract

**Background:**

Cancer has become increasingly prevalent in China over the past few decades. Among the factors that determine the quality of life of cancer patients, pain has commonly been recognized as a most critical one; it could also lead to the ineffective treatment of the cancer. Driven by the need for better pain management for cancer patients, our research team developed a mobile-based Intelligent Pain Management System (IPMS).

**Objective:**

Our objective was to design, develop, and test the IPMS to facilitate real-time pain recording and timely intervention among cancer patients with pain. The system’s usability, feasibility, compliance, and satisfaction were also assessed.

**Methods:**

A sample of 46 patients with cancer pain symptoms were recruited at the Oncology Center of Xinhua Hospital affiliated to Shanghai Jiao Tong University School of Medicine, Chongming Branch (hereinafter referred to as “the Oncology Center”). In a pretest, participants completed a pain management knowledge questionnaire and were evaluated using the baseline cancer pain assessment and Karnofsky Performance Status (KPS) evaluation. The participants were then randomly assigned into two groups (the trial group and the control group). After a 14-day trial period, another round of cancer pain assessment, KPS evaluation and pain management knowledge assessment were repeated. In the trial group, the data were fully automatically collected by the IPMS. In the control group, the data were collected using conventional methods, such as phone interviews or door-to-door visits by physicians. The participants were also asked to complete a satisfaction questionnaire on the use of the IPMS.

**Results:**

All participants successfully completed the trial. First, the feasibility of IPMS by observing the number of daily pain assessments recorded among patients was assessed. Second, the users’ satisfaction, effectiveness of pain management, and changes in the quality of their lives were evaluated. All the participants gave high satisfaction score after they used IMPS. Both groups reported similar pain scores and KPS scores at the baseline. At the end of the trial, the mean pain score of the trial group was significantly lower than of the control group (*P*<.001). The ending KPS score of the trial group was significantly higher than of the control group (*P*<.001). The improvement of pain management knowledge score in the trial group was more pronounced than that in the control group (*P*<.001).

**Conclusions:**

This study provided preliminary data to support the potentials of using IPMS in cancer pain communication between patients and doctors and to provide real-time supportive intervention on a convenient basis at a low cost. Overall, the IPMS can serve as a reliable and effective approach to control cancer pain and improve quality of life for patients with cancer pain.

**Trial Registration:**

Clinicaltrials.gov NCT02765269; http://clinicaltrials.gov/ct2/show/NCT02765269 (Archived by WebCite at http://www.webcitation.org/6rnwsgDgv)

## Introduction

### Status of Cancer and Pain Management in China

Cancer has become a leading cause of human death globally. The World Health Organization (WHO) estimated that cancer resulted in 8.2 million deaths in 2012 [1]. In China, the diagnosis rate of cancer soared over the past few decades as the country’s economy boomed rapidly. Statistical data show that China reported approximately 3.07 million new cases of diagnosed cancer in 2012, accounting for 21.8% of the global total [2-4]. Due to the novel diagnostic methods and therapeutic drugs, cancer patients have increased life expectancy than before. For doctors and other caregivers, maintaining cancer patients’ quality of life becomes increasingly important and challenging.

Research indicates that over one-third of cancer patients experience cancer pain, and this is known to be a major reason for lower quality of life for the patients [5,6]. The undertreatment of cancer pain is a worldwide problem [7,8]. To manage and further mitigate cancer pain, accurate and precise assessment is key [9-12]. An appropriately designed pain management system, if well planned and implemented, may effectively alleviate the pain of cancer patients. Obstacles preventing proper pain assessment include the lack of validated multidimensional tool to describe pain (intensity, quality, and location of the pain) and to evaluate pain interferences (emotional effects and daily activities) [12-15]. Conventional paper-based self-reported methods have major drawbacks including inaccuracies and biases. However, an advanced real-time pain assessment mechanism and electronic reporting system were found to be more effective in capturing pain data [16-20].

Another problem that China is facing is a wide resource gap across age groups and geographical areas. China is on its way to an aged and eventually super-aged nation in an accelerated pace on account of its economic growth and longstanding population policy. An increasing number of elderly people in cities and rural areas are in need of medical attention. Unlike many developed countries in the western hemisphere, China has very limited medical resources to cure and care for cancer patients in the underdeveloped rural areas. There is a critical shortage of medical doctors, nurses, and other medical professionals in China. A 2011 study shows that China has the doctor-to-resident ratio of 2.8 doctors per 1000 urban residents, whereas in the rural areas such ratio declines to 0.95 doctors per 1000 rural residents [21]. Therefore, home care and mobile care are believed to be future solutions to bridge the gap of the availability of medical resources between China’s urban and underdeveloped rural areas. Mobile care, based on mobile phones, is drawing attention because of the advantages of easy access, low cost, and quick response to patients’ needs.

### Description of Intelligent Pain Management System

Intelligent Pain Management System (IPMS) is a low cost, conveniently implemented system to facilitate real-time pain recording and timely intervention among Chinese cancer patients. This system has multiple features relying on mobile phones to evaluate real-time pain and KPS scores [22] for quality of life and to generate an action plan to visit the physician or to adjust pain medication dosage when the pain threshold is reached. During treatment, the IPMS may not only be used to evaluate patients’ pain status (self-management) but also be used to determine when a patient needs to adjust pain medication by the physician. According to the pilot study (2015), we found that the patients who use the IMPS had significantly more pain-undercontrol days compared with the control group [23]. In this study, we aim to further test the feasibility of IMPS, the usage satisfaction, and quality of life in cancer patients.

## Methods

### Design of Intelligent Pain Management System (IPMS)

**Figure 1:**
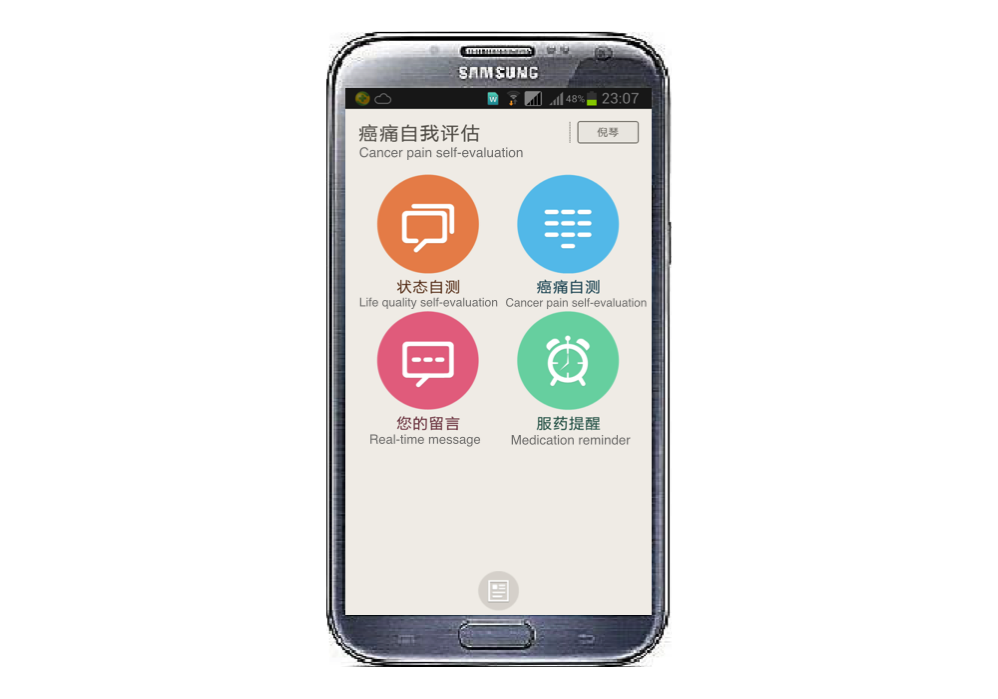
Screenshot of intelligent pain management system (IPMS) home screens: Life quality self-evaluation (upper left), cancer-pain self-evaluation (upper right), real-time message (lower left) and medication reminder (lower right).

The IPMS used in our research was designed to operate on the Android mobile operating system, in order to provide an affordable, portable, and easy to use environment for patients. The system design was performed by the Xinhua Translational Institute for Cancer Pain in Shanghai, China (hereinafter referred to as the Translational Institute). The design team adopted a modularity approach consisting of several functional subsystems to facilitate speedy development by multiple teams. The core system consisted of four modules: Life Quality Self-evaluation, Cancer Pain Self-evaluation, Real-time Messaging, and Standard Medication (Figure 1). After system architecture design, engineering work such as programming and system integration were outsourced to a professional IT company to produce an executable application on mobile phones.

**Figure 2:**
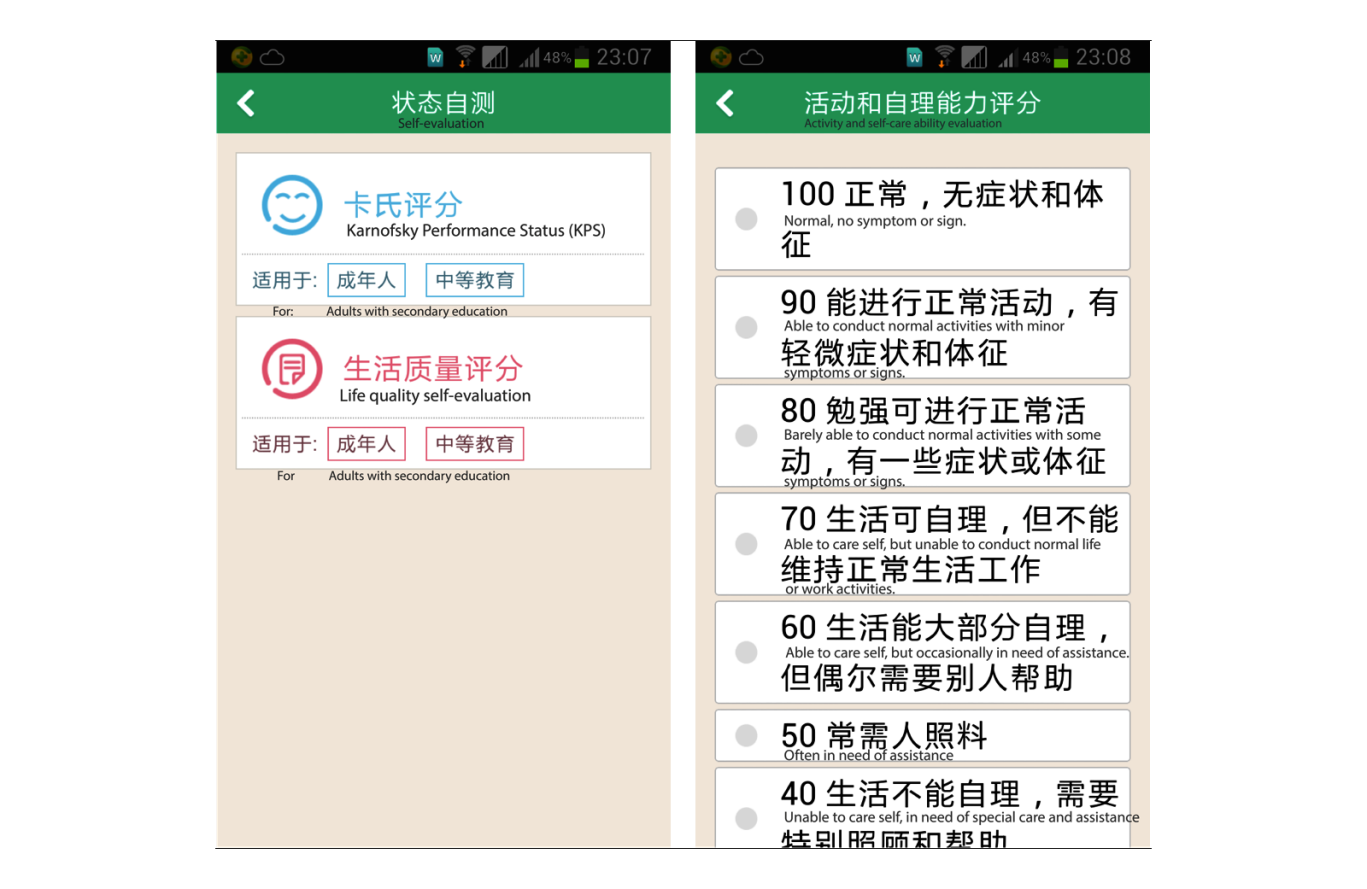
Screenshot of Karnofsky Performance Status (KPS) life quality self-evaluation module.

**Figure 3:**
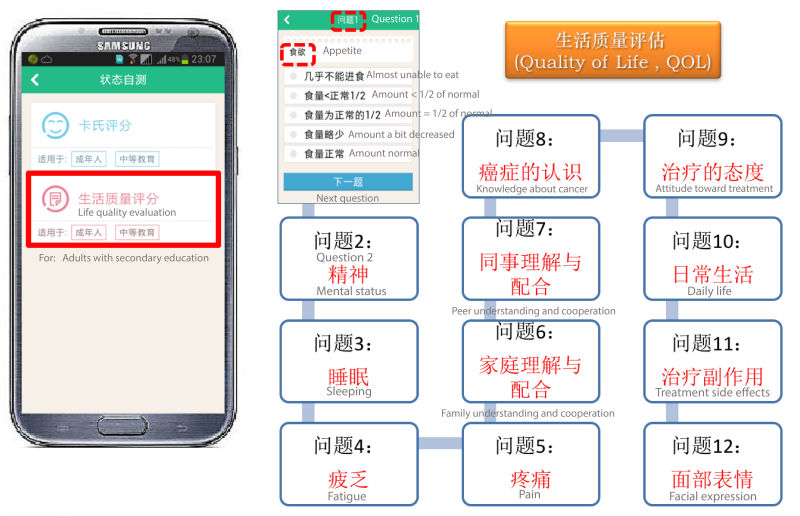
Questionnaire flow chart of lifer quality self-evaluation in life quality self-evaluation module.

#### Life Quality Self-Evaluation

This module consisted of two questionnaires. A KPS questionnaire was used to obtain the KPS score (Figure 2) and another 12-question questionnaire in a flow chart format was used to evaluate Quality of Life (QOL) scores (Figure 3).

#### Cancer Pain Self-Evaluation

**Figure 4:**
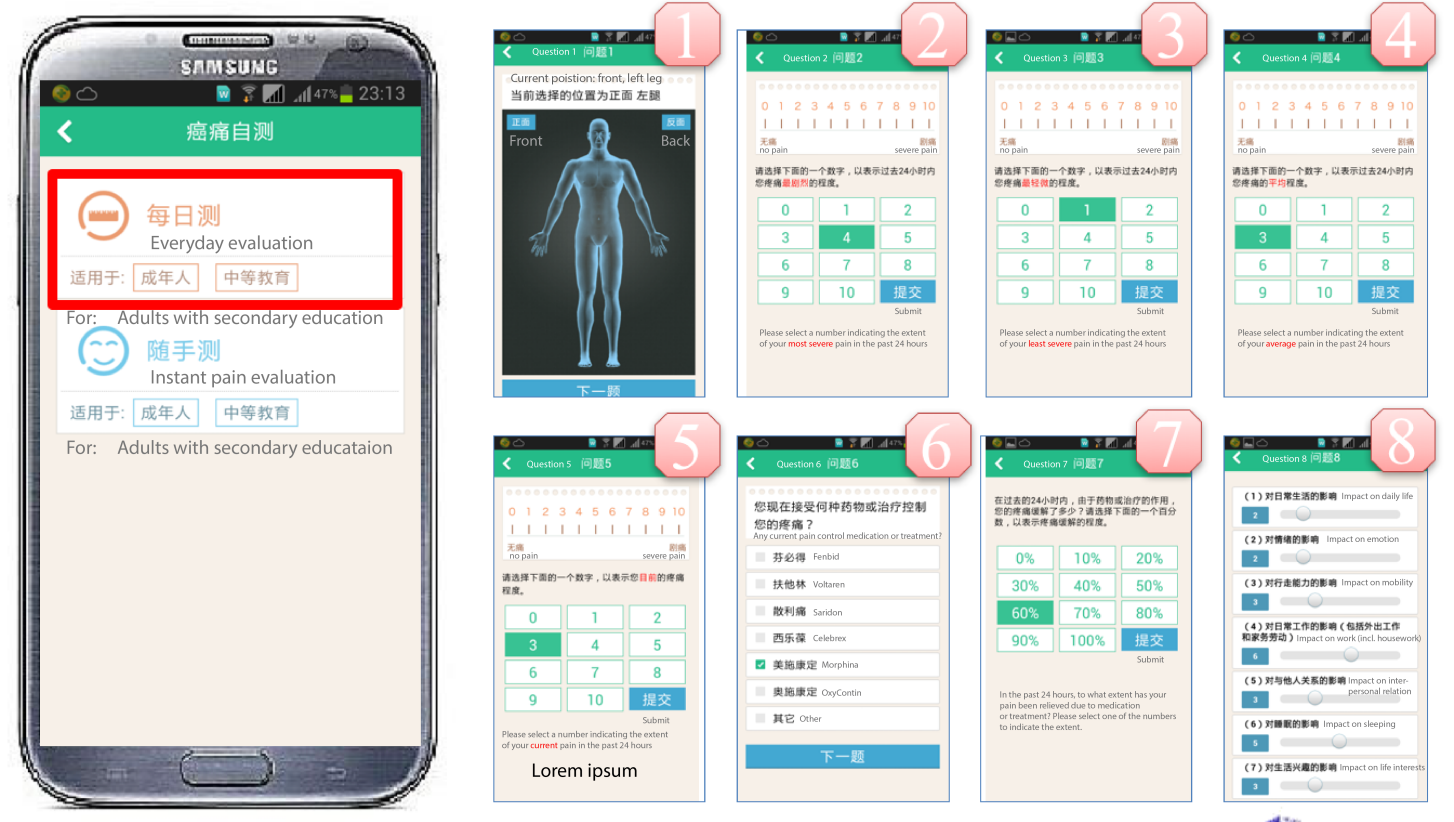
Screenshots of daily pain assess sub-module in cancer-pain self-evaluation module.

This core module of IPMS was designed to track patients’ self-reported pain data. It contained two submodules: a daily pain assessment submodule and an instant pain assessment submodule. The daily pain assessment submodule (Figure 4) displayed a body map on the smart phone screen allowing the patient to choose the precise position of a recently occurred cancer pain. The pain assessment questionnaire was developed based on the numerical rating scale (NRS) from 1 to 10 as an assessment vehicle. The patients were asked to identify the most, least, and average pains using NRS scores for the previous 24 hours and report the current pain score.

**Figure 5:**
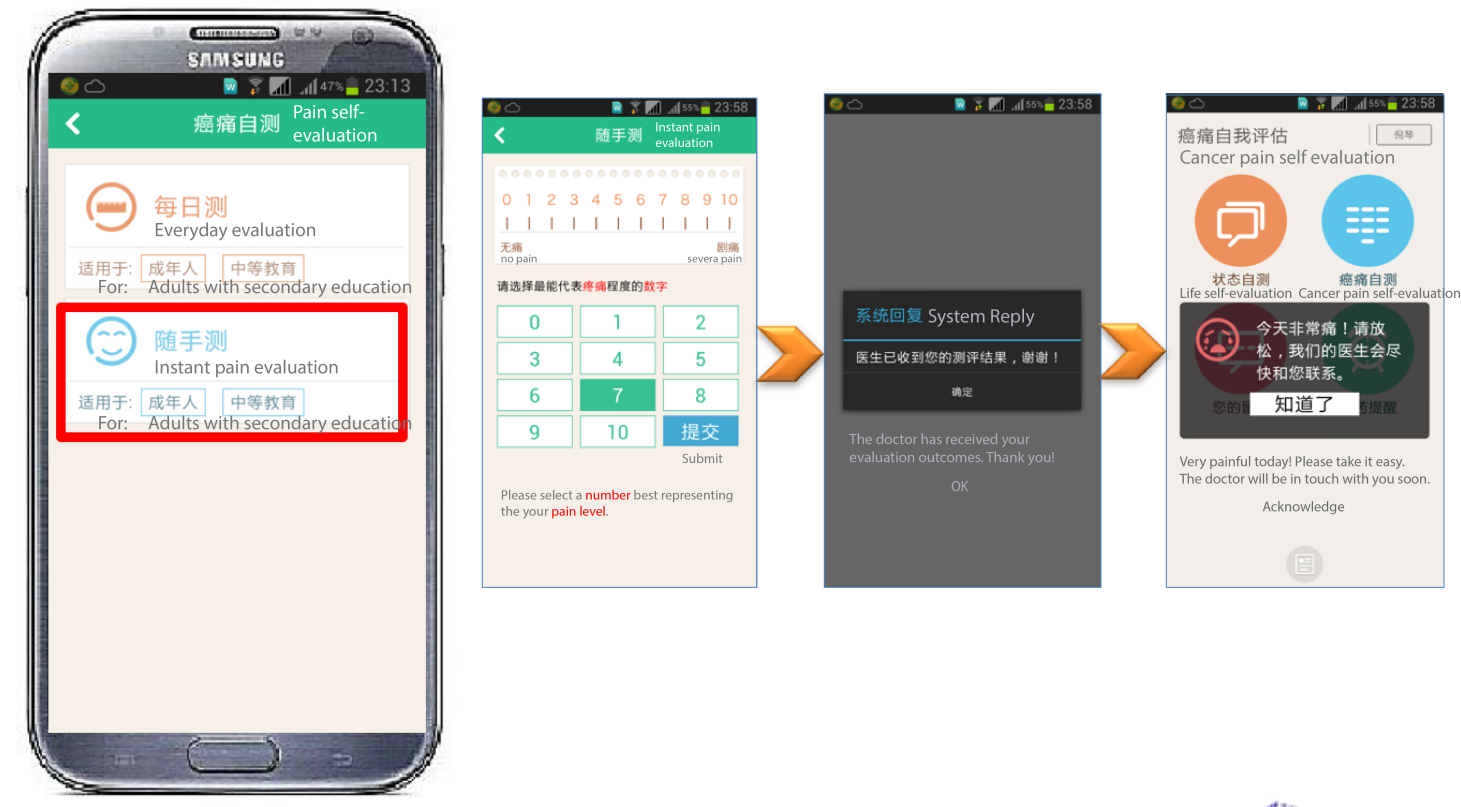
Screenshots of instant pain assess sub-module in cancer-pain self-evaluation module.

In addition, a list of pain medications was displayed to allow the patients to report their medications and their effectiveness. Lastly, a final pain assessment questionnaire consisted of 8 aspects (14 questions) to investigate other influences of cancer pain in their daily life, such as movement, hobbies, and relationships with family members, was administered. In the instant pain assessment submodule (Figure 5), NRS was used to evaluate the patient’s pain scores. The interface was designed to be user friendly for patients who suffered from variable intense pain (breakthrough pain). In this section, if a patient’s self-evaluated pain score reached a high level (>7), an automated message would be sent to the patient that he or she will be contacted by a physician soon.

#### Real-Time Messaging

**Figure 6:**
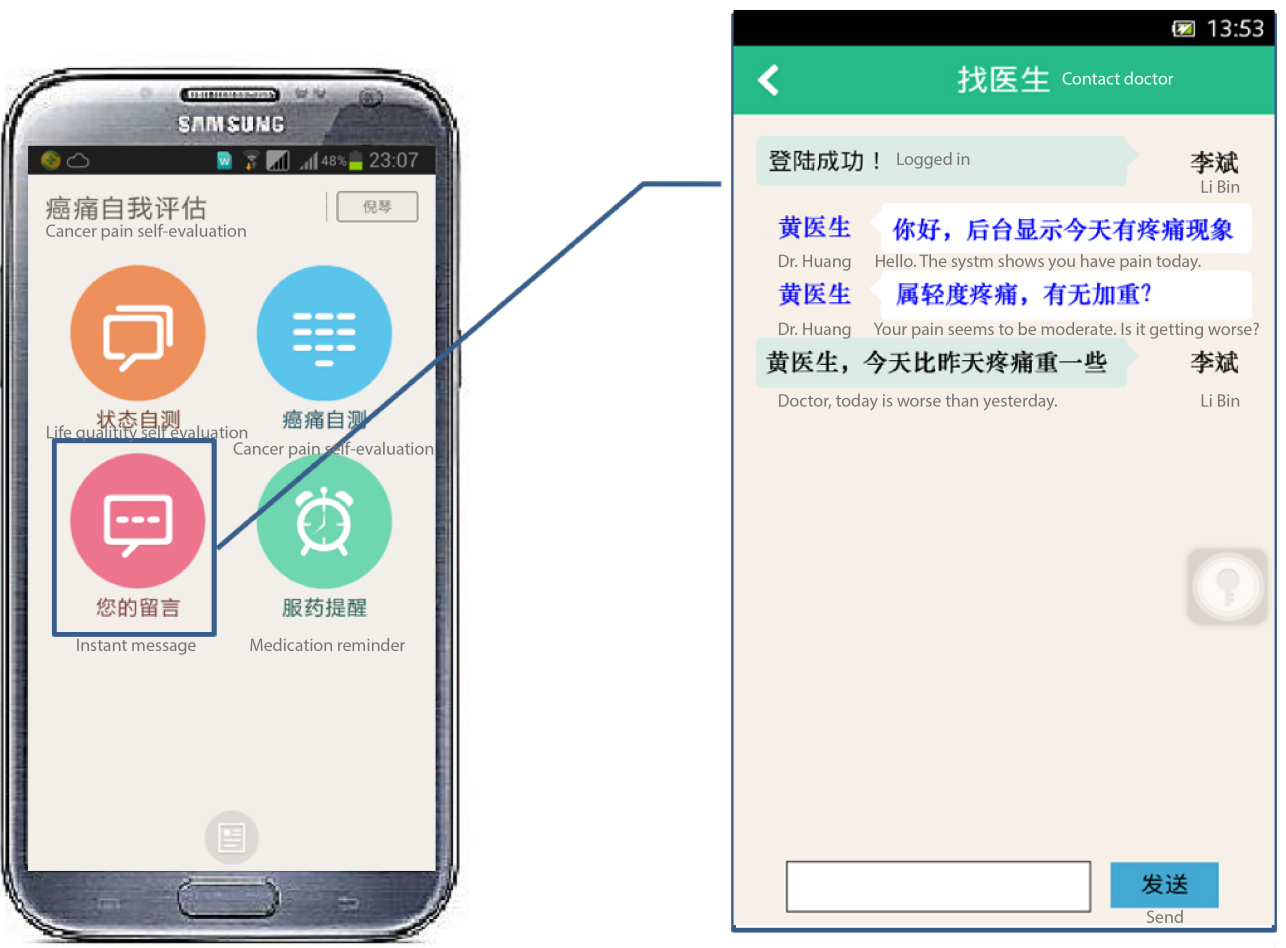
Screenshots of real-time message (left) and example (right).

This module was designed to assist patients to initiate a real-time consultation session on pain management with the doctors (Figure 6).

#### Standard Medication

**Figure 7:**
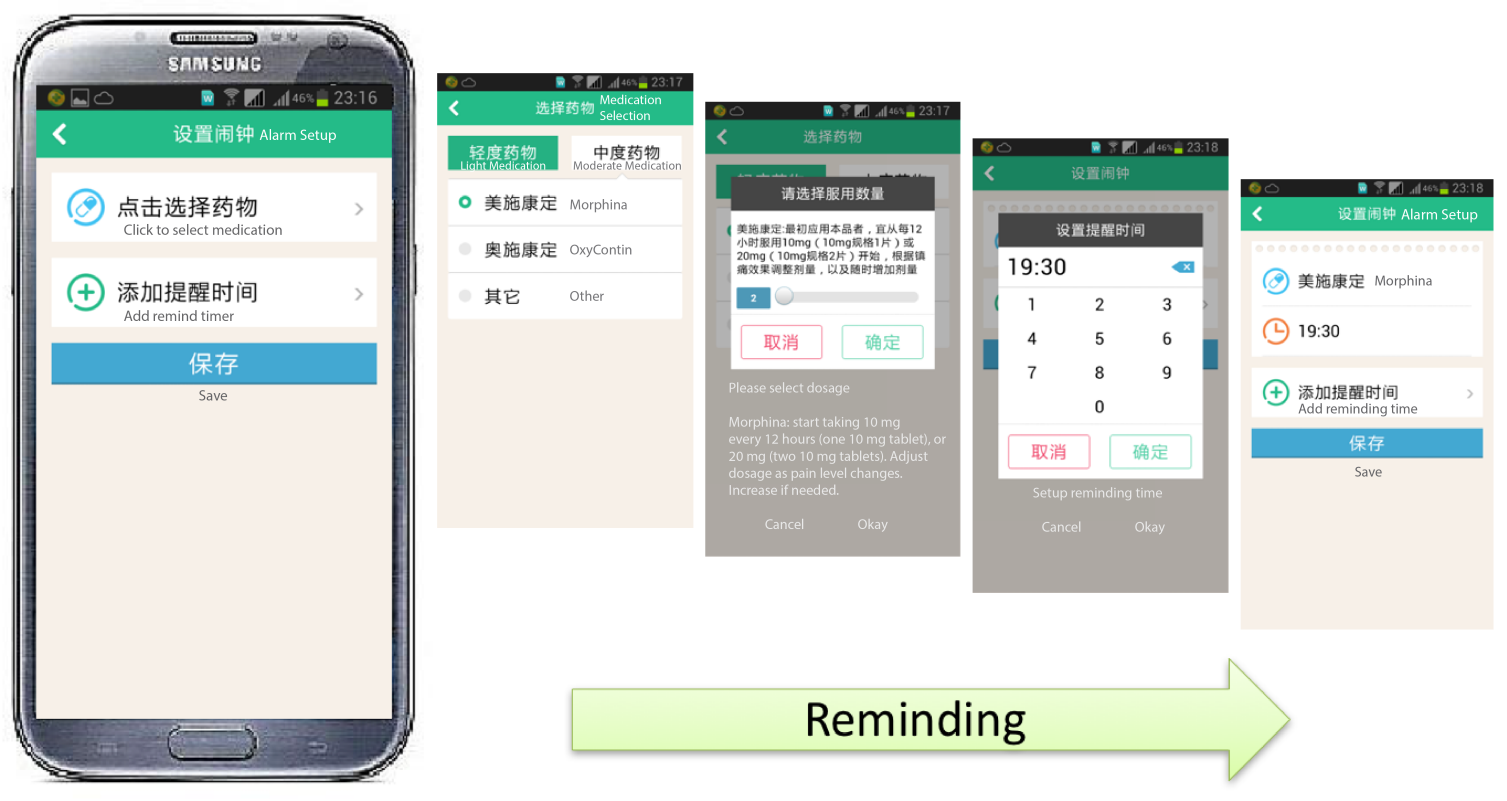
Screenshots of standard medication reminder module.

This module was designed to remind patients of their medication schedule (Figure 7) so that they would be assured to take the pain medicine on a regular basis.

### Study Design

An experiment was designed to test the effectiveness of IPMS on cancer pain management. The experiment involved two groups: an IMPS trial group and a control group.

#### IPMS Trial Group

The participants in the trial group were asked to complete a first day’s pain assessment questionnaire and the quality of life questionnaire on the mobile phones provided to them. Participants were then encouraged to use the IPMS as much as possible to record their pain status at least once every day for 14 days. They were asked to report the pain scores through the IPMS only. All other measurements were conducted by self-report questionnaires without face-to-face assessments.

#### Control Group

The control group was reached through conventional telephone calls or door-to-door visits to collect pain assessment data on a daily basis for 14 days.

### Principle Objectives

The primary objective was to assess the feasibility of the IPMS by observing the number of daily pain assessments recorded among the cancer patients.

The secondary objective was to evaluate the effectiveness of pain management, changes in the quality of their lives, and users’ satisfaction with IPMS app.

### Measurement

The IPMS satisfaction evaluation questionnaire was completed by participants at the end of the study. Each questionnaire involved multiple 5-point Likert scores with ratings associated with the options “extremely like it” (5 points), “like it” (4 points), “okay” (3 points), “dislike it” (2 points), and “extremely dislike it” (1 point). The data generated after the survey were used to evaluate the satisfaction of IPMS usage. The questionnaire also contained an open-ended question where participants were encouraged to give any other suggestions about IPMS they felt needed improvement.

A baseline pain assessment and a KPS evaluation were conducted using numerical rating scale (NRS) in both groups. After obtaining the consent, nurses conducted a standardized education session using a booklet to teach the participants proper pain-related and rating system knowledge. At the end of the trial, the pain assessment and KPS evaluation were repeated in both groups.

### Pain Management Knowledge, Pain Assessment, and Karnofsky Performance Status (KPS) Questionnaires

All participates were asked to complete a general information questionnaire on pain management containing five questions (Table 1) upon registration. Each question was a 3-point Likert type response anchored from “well-known,” “known,” to “unknown.” The same questionnaire was repeated at the end of the trial. Data generated at the beginning and the end of the trial were used to evaluate the change in participants’ pain knowledge.

**Table 1:** Pain management knowledge questionnaire.

Number	Question
1.	Do you know standard pain management?
2.	Do you know three step”ladder” cancer pain relief?
3.	Do you know methods other than three step “ladder”cancer pain relief
4.	Do you satisfy with your current pain management
5.	Do you feel confident about your pain management

## Results

This study was registered online at ClinicalTrials.gov (identifier: NCT02765269, Intelligent Pain Management System for Assessing Pain in Cancer Patients). With ethical review approved by the Medical Ethics Committee of Xinhua Hospital Affiliated to Shanghai Jiao Tong University School of Medicine, Chongming Branch, a randomized, controlled IPMS trial was conducted at the Oncology Center of Xinhua Hospital affiliated to Shanghai Jiao Tong University School of Medicine, Chongming Branch (hereinafter referred to as “the Oncology Center”). The study lasted from April 2016 to May 2016 in the Oncology Center with no changes to the study design and methods. The random allocation processes, how to enroll participants, as well as how to assign participants to intervention, were determined by physicians.

### Enrolment of the Participants

An original sample of 60 patients was recruited at office visits or in hospital visits in the center by physicians. The participating patients met the following screening criteria: (1) the patient was able to read Chinese and use a mobile phone; (2) the patient was aged between 45 and 70 years; (3) the patient was diagnosed with cancer and had self-reported cancer pain within a month prior to the study; (4) the patient was being seen on a regular basis by the oncology team; (5) the patient was under standard analgesia treatments; (6) the patient was estimated to have over 3 months survival time. Patients who self-reported to have severe cognitive impairments or major comorbid illnesses that would interfere with pain assessment were excluded from the experiment. For example, patients who received radiation therapy were excluded due to possible burning pain from the therapy. In April 2016, a total of 46 qualified cancer patients (14 females and 32 males) were finally included in this study.

### Participant Characteristics

Included participants were randomly allocated on a 1 to 1 ratio to either the IMPS trial group or the control group. Random allocation (simple randomization) of the participants was automatically performed using the random number table.

All participants were then randomly assigned into two groups: an IPMS trial group (25) and a control group (21). The trial group had 6 (24%) females and the control group had 8 (38%) females. The participants’ demographic information as well as their disease characteristics were summarized in Table 2.

Each participant in the trial group was provided an Android mobile phone with the IPMS loaded free of charge. They were given a demonstration and training by nurses and physicians on how to operate the smart phone and the IPMS app.

**Table 2:** Demographics and disease characteristics of patients.

Characteristics		IPMS	Control
Age (year)		67	68
Sex F/M, n		6/19	8/13
**Primary diagnosis, n (%)**				
	Lung cancer	8 (32)	9 (43)
	Column cancer	3 (12)	2 (9)
	Hepatic carcinoma	4 (16)	1 (4)
	Pancreatic cancer	2 (8)	2 (9)
	Stomach cancer	1 (4)	4 (19)
	Esophagus cancer	2 (8)	N/A^a^
	Breast cancer	1 (4)	N/A
	Ovary cancer	1 (4)	N/A
	Kidney cancer	1 (4)	1 (4)
	Osteocarcinoma	2 (8)	1 (4)

^a^N/A: not available.

### Data Analysis

The outcome assessor was blinded to the data collection. The collected data were analyzed using the R Statistical Software Package 3.1.3. Independent Student’s *t*-test and the Chi-square (χ^2^) test were used to analyze the differences (NRS, AGE, KPS) in pain controlled duration and breakthrough pain between the trial group and the control group. The significant difference was determined by *P*<.05.

### IPMS Feasibility Testing

The compliance rate was consistently high over the course of the trial with no statistical difference in total number of daily pain assessment between week 1 and week 2 (16.72 [SD 5.95] vs 18.36 [SD 6.35], *P*>.05). Further analyses showed that the total number of pain assessments between the day times and the night times was 1.82 (SD 0.43) vs 0.56 (SD 0.18), respectively. In addition, there was no significant difference between the usage times of weekdays and weekends (2.45 [SD 0.6] vs 2.15 [SD 0.18], *P*>.05).

### Pain Management and KPS Evaluation

At the beginning of the trial, there was no significant difference in the baseline pain scores (3.28 [SD 0.68] vs 2.90 [SD 0.62], *P*>.05) between the two groups. Over the 14-day trial period, the average pain score of the trial group was 2.53 (SD 0.42), compared with 2.81 (SD 0.47) of the control group with a significant difference (*P*<.001). At the end of the trial period, the average pain score of the trial group was 2.20 (SD 0.50), compared with 2.95 (SD 0.59) of control group with a significant difference between the two groups, *P*<.001 (Table 3).

As to the evaluation of the acquired pain management knowledge after 14 day’s IPMS interaction, there was a 2.96 (SD 0.61) increase in the knowledge score of the trial group after using the IPMS for two weeks, compared with a 0.81 (SD 0.67) increase of the control group (*P*<.001). Although both groups demonstrated increased pain management knowledge, the IPMS trial group indicated a higher score increase in pain management knowledge than did the control group.

Another application of the IPMS was the education and evaluation of quality of life through KPS scores. The baseline KPS scores in the IPMS trial group were no different than those of the control group (50.80 [SD 7.02] vs 50.95 [SD 7.40], *P*=.94) before the participants had entered into the trial. At the end of the trial, the KPS was re-evaluated in the two groups (68.80 [SD 7.23] vs 56.19 [SD 7.40], *P*<.001). Both groups increased mean KPS significantly from the baseline, but the mean increase in the IPMS trial group was significantly larger than the mean increase in the control group (16.15 [SD 7.68] vs 5.23 [SD 5.11], *P*<.01).

**Table 3:** Pain management and KPS score comparisons between IPMS and control groups.

Scores		IMPS			Control				Group difference		
		Baseline	Day 14	Change from baseline		Baseline	Day 14	Change from baseline		Baseline	Change from baseline
Pain evaluation												
	Mean (SD)	3.28 (0.68)	2.20 (0.50)			2.90 (0.62)	2.95 (0.59)				
	*P* value			<.001				.58		.06	<.001
Pain knowledge management												
	Mean (SD)	5.16 (0.75)	8.12 (0.07)			4.09 (0.83)	4.90 (1.09)				
	*P* value			<.001				.009			<.001
KPS												
	Mean (SD)	50.80 (7.02)	68.80 (7.23)			50.95 (7.40)	56.19 (7.40)				
	*P* value			<.001				.023		.94	<.001

### Satisfaction

A posttrial evaluation was conducted to measure the participants’ satisfaction towards the IPMS. On the ease of use of the IPMS, out of the 25 participants in the trial group, 9 (36%) indicated “very much like it (the IPMS)” and 16 (64%) indicated “like it (the IPMS).” No participant indicated dislike of the IPMS. On the helpfulness of the IPMS, 20 (80%) responded “very helpful” and 5 (20%) responded “helpful.” On the software technical support, 18 (72%) indicated “very much like it” and 7 (28%) indicated “like it.” On the consultant and training course, a majority of participants 18 (72%) reported “very much like it” and (7) 28% reported “like it.” On the prompt response for help, 7 (28%) indicated “very much like it,” 11 (44%) indicated “like it,” and 7 (28%) indicated “okay.” The average score of each question is shown in Table 4. The data suggests a high level of user satisfaction towards IPMS.

**Table 4:** Usability and satisfaction after 2 weeks (n=25). The rate was 1 (extremely dislike it) to 5 (extremely like it).

Number	Question	Mean (SD)
1	The convenience to use IPMS	4.46 (0.49)
2	Do you think IPMS is helpful to your pain management?	3.92 (0.61)
3	How do you like IPMS	4.30 (0.46)
4	Software technical support	4.73 (0.44)
5	Consultant and training course	4.65 (0.47)
6	Prompt response for help	4.53 (0.49)

## Discussion

### Principal Findings

This research involved a cohort of cancer patients with cancer pain in a study of the usability and effectiveness of an IPMS system designed for this study. We presented data on the compliance, satisfaction, evaluation of pain management in a 2-week clinical trial. Overall, the study demonstrated that the IPMS gained a high rate of compliance and satisfaction among the participants. With little or no additional clinical intervention, IPMS had the potential to improve pain management and quality of life for cancer patients with cancer pain.

### Comparison With Prior Work

To the best of our knowledge, this study could be the first to report on the development, usability testing, and evaluation of cancer pain and KPS with an intelligent pain management system through mobile applications in China. A search of the literature has yielded several reports on the assessment of usability of technology-based interventions on cancer pain. However, majority of past research were based on telephone interventions to connect patients with health providers [4,24]. Stinson group reported the development and testing of a multidimensional iPhone (R) pain assessment application for youth cancer patients. Their application was a game-based program to assess pain, which was specially designed for adolescent cancer patients and with no intervention [25,26].

This study implemented the telephone-based intervention as the control group to compare with the mobile Internet-based IPMS. The IPMS was developed as a multidimensional tool not only for real-time pain assessment but also for KPS evaluation, medication reminder, real-time messaging consultation, and pain management education. Such mechanism was able to convey clinical assistance and intervention between the health providers and the patients in an efficient and effective way. It was the real-time messaging that mattered to the patients’ pain management. The IPMS allowed patients to be able to instantaneously assess and report pain, thus the doctors were able to provide prompt advice on the dosage change of pain control medication, which was not possible in traditional ways. The rapid adoption of mobile devices in China provided a promising future of the IPMS in cities and rural areas. We are expecting that the IPMS will become a popular communicating vehicle between physicians and cancer patients in the near future.

### Strengths and Limitations

The clinical trial yielded a perfect compliance rate (100%) of the IPMS use regardless of time—day times or night times, weekdays, or weekends. The compliance rate was consistent over the course of the trial with no significant difference between week 1 and week 2. The slightly heavier usage at the night times highlighted the potential of IPMS, as obtaining the pain data at night times was always a challenge through conventional methods. Real-time pain satisfaction assessment data have been valuable for researchers and care providers. With a powerful tool as the IPMS, we may be able to better understand the pain burst out patterns and provide timely response to the patients’ need, allowing for improved pain management and higher quality of pain treatment.

Through the usability test, we were able to test the user interface and the basic functions of the IPMS. The patient satisfaction rate was high for its easy use, as well as being helpful for the consultant and training courses. However, there was still room for improvement in terms of prompt response for help. As this is the first smart phone app for pain management for both patients and healthcare professionals to use, it may take time for both parties to get used to the system. A clinical study of longer duration will be conducted to address this question in the near future.

In this study, the improvement of cancer pain management was more pronounced in the IPMS trial group than in the control group, which suggests that IPMS is beneficial. Testing data also revealed that the knowledge of pain management and quality of life were all significantly increased in the IPMS group compared with the control group. Patients in both groups were exposed to same levels of clinical care, educational program, and pain management knowledge training. The reason the IPMS group had more benefit, may be due to the interactive-feedback learning mechanism that gave the trial group patients more confidence and knowledge to deal with pain management. A similar phenomenon has been observed and reported by Liu LF group in the literature [17].

This study had several limitations that may tamper our results. First, the duration of the trial period was relatively short and the sample size was relatively small. Due to the limited sample size, the distribution of cancers types was not balanced between groups as certain types of cancer had only 1 patient in the sample. The clinical trial group was drawn from a patient pool with different stages of cancer, which may limit the generalizability of this study. Future study is warranted to test the effectiveness of IPMS with a larger sample of participants and longer time period with improved randomization and balance.

### Conclusions

In conclusion, this study underscored the feasibility and acceptability of IPMS as a novel and effective pain assessment tool for patients with cancer pain. Participants found the system easy to use and helpful. IPMS was found to be beneficial for pain management, quality of life, and pain management education in a 14 days’ clinical trial. It is believed that IPMS has the potential to change the current atlas of pain management in China, especially in underdeveloped rural areas with improved efficiency and effectiveness of pain management and interactions between cancer patients and health professionals.

## Appendix

Multimedia Appendix 1 The trial is reported in accordance with CONSORT-eHEALTH.

## Abbreviations

IPMS: intelligent pain management system
KPS: Karnofsky Performance Status
NRS: numerical rating scale

## References

[1] World Health Organization (2017). WHO.

[2] Edwards BK, Noone A, Mariotto AB, Simard EP, Boscoe FP, Henley SJ, Jemal A, Cho H, Anderson RN, Kohler BA, Eheman CR, Ward EM (2014). Annual report to the Nation on the status of cancer, 1975-2010, featuring prevalence of comorbidity and impact on survival among persons with lung, colorectal, breast, or prostate cancer. Cancer.

[3] Goss PE, Strasser-Weippl K, Lee-Bychkovsky BL, Fan L, Li J, Chavarri-Guerra Y, Liedke PE, Pramesh CS, Badovinac-Crnjevic T, Sheikine Y, Chen Z, Qiao Y, Shao Z, Wu Y, Fan D, Chow LW, Wang J, Zhang Q, Yu S, Shen G, He J, Purushotham A, Sullivan R, Badwe R, Banavali SD, Nair R, Kumar L, Parikh P, Subramanian S, Chaturvedi P, Iyer S, Shastri SS, Digumarti R, Soto-Perez-de-Celis E, Adilbay D, Semiglazov V, Orlov S, Kaidarova D, Tsimafeyeu I, Tatishchev S, Danishevskiy KD, Hurlbert M, Vail C, St LJ, Chan A (2014). Challenges to effective cancer control in China, India, and Russia. Lancet Oncol.

[4] Agboola SO, Ju W, Elfiky A, Kvedar JC, Jethwani K (2015). The effect of technology-based interventions on pain, depression, and quality of life in patients with cancer: a systematic review of randomized controlled trials. J Med Internet Res.

[5] Lesage P, Portenoy RK (1999). Trends in cancer pain management. Cancer Control.

[6] Portenoy RK, Lesage P (1999). Management of cancer pain. Lancet.

[7] Deandrea S, Montanari M, Moja L, Apolone G (2008). Prevalence of undertreatment in cancer pain. A review of published literature. Ann Oncol.

[8] Greco MT, Roberto A, Corli O, Deandrea S, Bandieri E, Cavuto S, Apolone G (2014). Quality of cancer pain management: an update of a systematic review of undertreatment of patients with cancer. J Clin Oncol.

[9] Forbes K (2011). Pain in patients with cancer: the World Health Organization analgesic ladder and beyond. Clin Oncol (R Coll Radiol).

[10] Kwon JH (2014). Overcoming barriers in cancer pain management. J Clin Oncol.

[11] Jacobsen R, Liubarskiene Z, Møldrup C, Christrup L, Sjøgren P, Samsanaviciene J (2009). Barriers to cancer pain management: a review of empirical research. Medicina (Kaunas).

[12] Hida S, Nishimura K, Nishio Y, Oishi K, Takeuchi H, Yoshida O (1991). VAB-6 chemotherapy causes spurious elevation of alpha-fetoprotein associated with liver dysfunctions. Urol Int.

[13] Dalal S, Bruera E (2012). Assessing cancer pain. Curr Pain Headache Rep.

[14] Palermo TM (2009). Assessment of chronic pain in children: current status and emerging topics. Pain Res Manag.

[15] Stinson JN, Kavanagh T, Yamada J, Gill N, Stevens B (2006). Systematic review of the psychometric properties, interpretability and feasibility of self-report pain intensity measures for use in clinical trials in children and adolescents. Pain.

[16] Stone AA, Broderick JE (2007). Real-time data collection for pain: appraisal and current status. Pain Med.

[17] Jan R, Wang J, Huang M, Tseng S, Su H, Liu L (2007). An internet-based interactive telemonitoring system for improving childhood asthma outcomes in Taiwan. Telemed J E Health.

[18] Mulvaney SA, Anders S, Smith AK, Pittel EJ, Johnson KB (2012). A pilot test of a tailored mobile and web-based diabetes messaging system for adolescents. J Telemed Telecare.

[19] Heinonen R, Luoto R, Lindfors P, Nygård C (2012). Usability and feasibility of mobile phone diaries in an experimental physical exercise study. Telemed J E Health.

[20] O'Reilly GA, Spruijt-Metz D (2013). Current mHealth technologies for physical activity assessment and promotion. Am J Prev Med.

[21] Watts J (2006). China's rural health reforms tackle entrenched inequalities. Lancet.

[22] Schag CC, Heinrich RL, Ganz PA (1984). Karnofsky performance status revisited: reliability, validity, and guidelines. J Clin Oncol.

[23] Weiwei Hu, Feng Jiang, Gang Ding (2015). The application of intelligent pain management system in cancer pain patients. Chin J Pain Med.

[24] Somers TJ, Abernethy AP, Edmond SN, Kelleher SA, Wren AA, Samsa GP, Keefe FJ (2015). A pilot study of a mobile health pain coping skills training protocol for patients with persistent cancer pain. J Pain Symptom Manage.

[25] Jibb LA, Stevens BJ, Nathan PC, Seto E, Cafazzo JA, Stinson JN (2014). A smartphone-based pain management app for adolescents with cancer: establishing system requirements and a pain care algorithm based on literature review, interviews, and consensus. JMIR Res Protoc.

[26] Stinson JN, Jibb LA, Nguyen C, Nathan PC, Maloney AM, Dupuis LL, Gerstle JT, Alman B, Hopyan S, Strahlendorf C, Portwine C, Johnston DL, Orr M (2013). Development and testing of a multidimensional iPhone pain assessment application for adolescents with cancer. J Med Internet Res.

